# A Patient Case of Malan Syndrome Involving 19p13.2 Deletion of *NFIX* with Longitudinal Follow-Up and Future Prospectives

**DOI:** 10.3390/jcm13216575

**Published:** 2024-11-01

**Authors:** Simran Makker, Bernadine R. Gagnon, Isabella Trew, Vivian Mougios, Anne Hanna, Jessica M. Cale, Craig S. McIntosh

**Affiliations:** 1 Laurel Springs School, Ojai, CA 93023, USA; simrannmakker@gmail.com; 2 Edward D. Mysak Clinic for Communication Disorders, Teachers College, Columbia University, New York, NY 10027, USA; bgagnon@tc.columbia.edu; 3 Centre for Molecular Medicine and Innovative Therapeutics, Health Futures Institute, Murdoch University, Murdoch, WA 6150, Australia; isabella.trew@murdoch.edu.au (I.T.); jessica.cale@murdoch.edu.au (J.M.C.); 4 Perron Institute for Neurological and Translational Science, Nedlands, WA 6009, Australia; 5 Action Potential Institute, 145 W 96th S, New York, NY 10025, USA; vivian@drmougios.com; 6 Gold Coast Optometric Vision Performance, Oyster Bay, NY 11771, USA; anniereuter@gmail.com

**Keywords:** Malan syndrome, *NFIX*, 19p13.2 deletion, neurodevelopmental delay, overgrowth disorder

## Abstract

**Background and Objectives:** Malan syndrome is a rare overgrowth syndrome resulting from *NFIX* haploinsufficiency due to heterozygous loss-of-function mutations or microdeletions of *NFIX* on chromosome 19 at p13.2. Phenotypic presentation can vary but is characterized by macrocephaly, long and slender body habitus, skeletal abnormalities, and intellectual disability. **Methods:** Here, we report on the presentation, management, and development of a patient with Malan syndrome, highlighting the clinical and behavioral aspects of this syndrome, therapeutic interventions employed, and the course of disease over a 15-year period. We review medical records, cytogenetic analysis and neuropsychologic testing results, as well as speech pathology, optometric, and medical reports. In addition, we discuss personalized therapeutic strategies that could potentially be exploited in the future for such overgrowth syndromes. **Results:** To our knowledge, this is the first longitudinal follow-up report of a case of Malan syndrome to highlight the clinical course, interventions employed, and resulting improvements in neurocognitive function over time. **Conclusions:** This case highlights the importance of early diagnosis, intervention, and preventative care in overgrowth syndromes, as well as the potential for therapeutic intervention in the future.

## 1. Introduction

Overgrowth syndromes present at birth and postnatally are characterized by macrocephaly, facial dysmorphia, intellectual disability, advanced bone age, and increased height. Such syndromes include Beckwith–Wiedemann syndrome, Sotos syndrome, Weaver syndrome, Simpson–Golabi–Behmel syndrome, Marshall–Smith syndrome [1], Proteus syndrome, and Bannayan–Riley–Ruvalcaba syndrome [2].

Nuclear factor I X-type (*NFIX*) belongs to the nuclear factor I (NFI) family of transcription factors that encode proteins with a conserved N-terminal DNA-binding/dimerization domain and a C-terminal transactivation/repression domain. NFI transcription factors act as homo- and hetero-dimers and bind with high affinity to the palindromic consensus sequence TTGGC(N5)GCCAA, and function as dual transcription activators or repressors based on interactions with dimerization partner(s), which can include other members of the NFI family [3,4,5,6,7].

In vertebrates, the NFI family consists of *NFIA*, *NFIB*, *NFIC*, and *NFIX* [1], which are broadly expressed in the developing and adult nervous systems. Murine *Nfix* is expressed throughout embryogenesis, with expression in embryonic mouse telencephalon [5], cerebellum, pontine nuclei, and spinal cords. *Nfix* -/- mice display significantly larger brains, evince aberrant postnatal cortical, ventricular, and hippocampal formation [8,9,10], delayed astrocyte differentiation [11], development of cerebellar granule neurons and Purkinje cells, and differentiation of mature cerebellar glia [12,13]. NFIX is broadly involved in hematopoiesis [14,15], regulates embryonic-to-fetal muscle transition [16] and myostatin expression [17], and is potentially implicated in oncogenesis of gliomas, squamous cell carcinoma, and colorectal cancer [7,18,19].

Malan syndrome (Online Mendelian Inheritance in Man [OMIM] #614753), previously known as Sotos syndrome 2, has an estimated prevalence of 1 per 1,000,000 persons [20]. It occurs due to *NFIX* haploinsufficiency resulting from heterozygous loss-of-function mutations or microdeletions of *NFIX* on chromosome 19 at p13.2 [20], most commonly in exon 2. *NFIX* mutations can arise as missense, nonsense, frameshift, in-frame deletion, or splicing.

Here, we report a case of Malan syndrome caused by a 75 kb heterozygous microdeletion on the short arm of chromosome 19 at p13.2 involving exons 3 to 11 of *NFIX*, the smallest microdeletion in this gene reported thus far. We discuss the patient’s clinical features as well as improvements in his neurocognitive function over time in the presence of intensive therapeutic support.

## 2. Detailed Case Description

**Figure 1:**
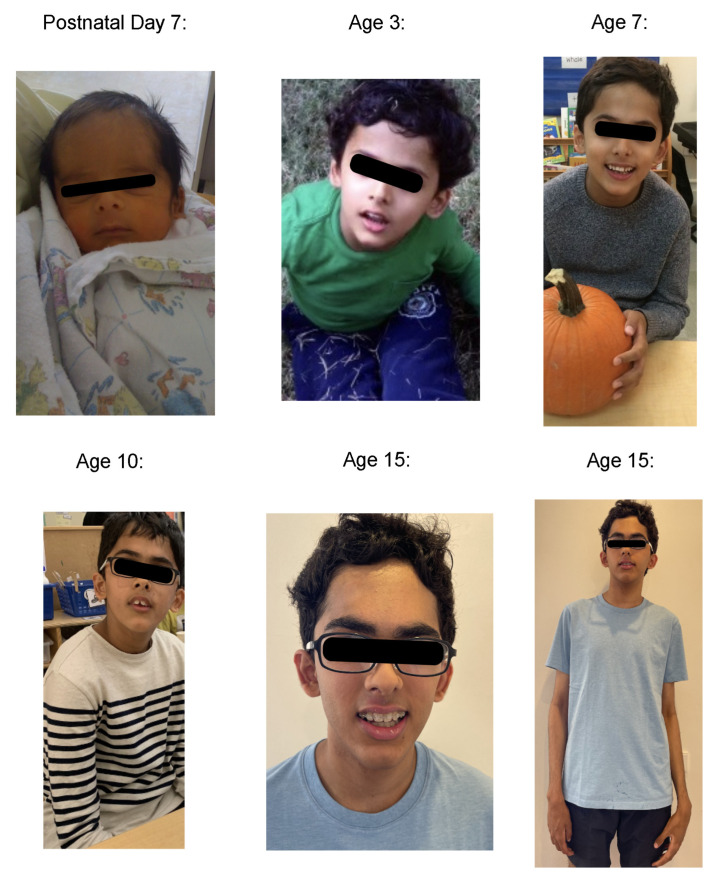
Facial photographs of the patient, by age.

The patient is the second child of nonconsanguineous parents, delivered by cesarean section at 36 weeks’ gestation due to a complete placenta previa pregnancy. Birth weight and length were 2.6 kg and 19 cm, respectively. Apgar scores were 6 and 9 at 1 and 5 min, respectively, at which point he became apneic with grunting, and was admitted to the NICU for continuous positive airway pressure (CPAP) and eventual intubation for hypoxemia and administration of surfactant. The patient had frontal bossing, deep-set eyes, a depressed nasal bridge, a small mouth (Figure 1), and a high-arched palate, and long fingers and toes with deep creases in the palms and soles. Hypotonia was noted, with good deep tendon reflexes. He was treated with prophylactic antibiotics, and blood cultures were negative. Hyperbilirubinemia with a peak bilirubin of 11.5 mg/dL was noted. Brain ultrasound noted a small germinal matrix hemorrhage on the left side but was otherwise normal. The patient was discharged from the NICU on day 10.

At 3 months of age, ophthalmologic examination revealed pseudoesotropia secondary to a broad nasal bridge and mild bilateral myopia with astigmatism. At 5.5 months, his head circumference was 46 cm (98th percentile) and he was diagnosed with moderate-to-severe deformational plagiocephaly, which was treated with cranial orthosis. At 8 months, the patient was noted to have elfin facies, with low-set ears and a high-arched palate, a small chin and nose (Figure 1), intact cranial nerves, normal motor tone, good grasp and deep tendon reflexes, prehensile grip, and mild motor delay. At 14 months, he was diagnosed with idiopathic inferior oblique muscle overaction and bilateral fourth nerve palsy, for which he underwent bilateral inferior oblique anterior transposition. The patient experienced frequent vomiting until the age of 15 months, otitis media infections until the age of 4 years, teeth overcrowding and malocclusion, which were treated with extractions and orthodontic care, and testicular torsion, which was treated with unilateral orchiectomy at 13 years of age.

**Figure 2:**
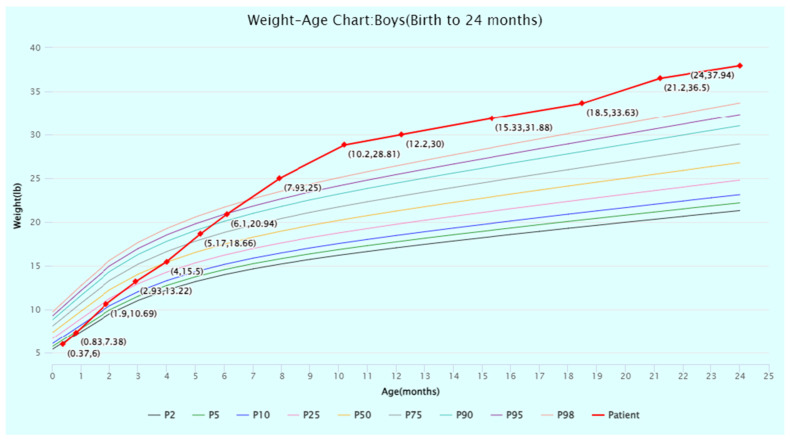
Weight-for-age percentiles for boys (birth to 24 months) from the World Health Organization (WHO) Child Growth Standards. The patient’s weight at timepoints from birth to 24 months of age is displayed and plotted (in red) against the WHO percentiles. Source: WHO Child Growth Standards (https://www.who.int/tools/child-growth-standards/standards (accessed on 1 July 2024)). Abbreviation: P, percentile.

**Figure 3:**
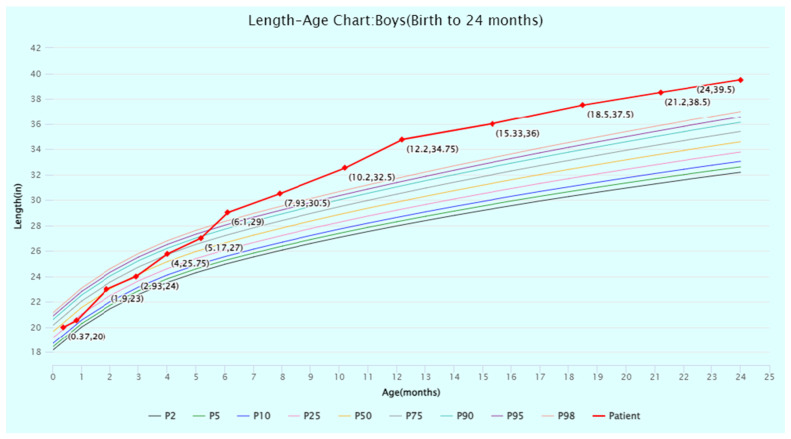
Length-for-age percentiles for boys (birth to 24 months) from the World Health Organization (WHO) Child Growth Standards. The patient’s length at timepoints from birth to 24 months of age is displayed and plotted (in red) against the WHO percentiles. Source: WHO Child Growth Standards (https://www.who.int/tools/child-growth-standards/standards (accessed on 1 July 2024)). Abbreviation: P, percentile.

**Figure 4:**
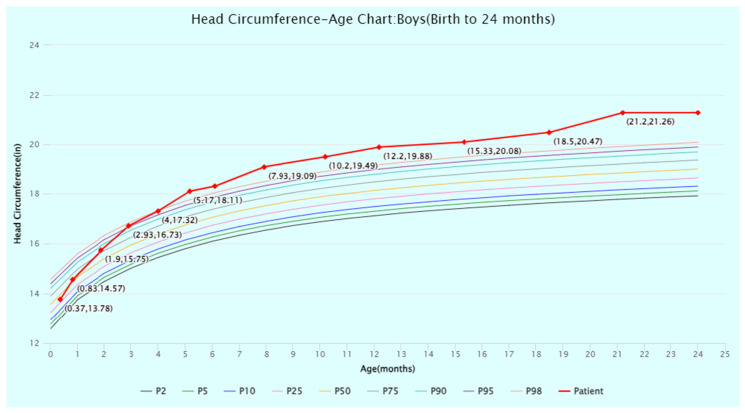
Head circumference-for-age percentiles for boys (birth to 24 months) from the World Health Organization (WHO) Child Growth Standards. The patient’s head circumference at timepoints from birth to 24 months of age is displayed and plotted (in red) against the WHO percentiles. Source: WHO Child Growth Standards (https://www.who.int/tools/child-growth-standards/standards (accessed on 1 July 2024)). Abbreviation: P, percentile.

**Figure 5:**
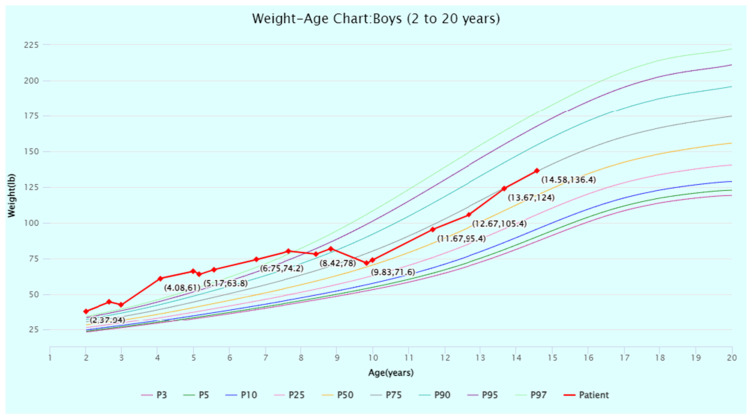
Weight-for-age percentiles for boys (2 to 20 years) from the Centers for Disease Control (CDC) Growth Charts. The patient’s weight at timepoints from 2 to 14 years of age is displayed and plotted (in red) against the CDC percentiles. Source: CDC Growth Charts (https://www.cdc.gov/growthcharts/cdc-growth-charts.htm (accessed on 1 July 2024)). Abbreviation: P, percentile.

**Figure 6:**
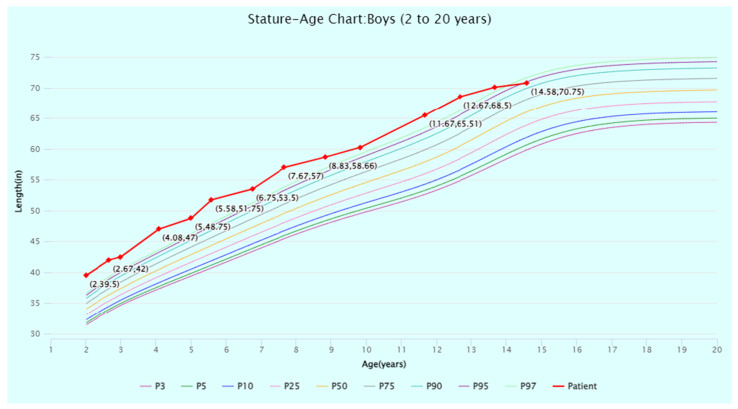
Stature-for-age percentiles for boys (2 to 20 years) from the Centers for Disease Control (CDC) Growth Charts. The patient’s stature at timepoints from 2 to 14 years of age is displayed and plotted (in red) against the CDC percentiles. Source: CDC Growth Charts (https://www.cdc.gov/growthcharts/cdc-growth-charts.htm (accessed on 1 July 2024)). Abbreviation: P, percentile.

The patient’s head circumference, weight, and height growth charts (Figure 2, Figure 3, Figure 4, Figure 5 and Figure 6) highlight the patient’s overgrowth in relation to head circumference and height. On the most recent clinical assessment at 14 years of age, the patient’s height was 1.8 m (94th percentile), weight was 61.4 kg (75th percentile), blood pressure was 120/62 mmHg, and complete blood count, liver and renal function, and vitamin D level were within normal limits. The patient was in Tanner stage 4, and no scoliosis was noted.

### 2.1. Gross Motor Development

The patient babbled at 3 months of age, smiled at 4 months, rolled at 8 months, sat without support at 10 months, waved at 12 months, sat independently at 12 months, said “dada”, specific to his father, at 19 months, crawled at 20 months, and walked independently by 21 months. Until the age of 19 months, he ate only pureed foods, and at 27 months, he drank from a straw and consumed solid/non-pureed foods. At 24 months, he was noted to have persistent hypersalivation.

### 2.2. Therapeutic Intervention

At 19 months of age, intensive rehabilitation was initiated, including occupational therapy (5 sessions per week), physical therapy (3 sessions per week), speech and feeding therapy (4 sessions per week), and cognitive behavioral therapy and special education instruction (4 sessions per week). Although no deficiencies were identified, Tri-Vi-Sol (1 mL daily), complete omega oil (0.25 tsp twice daily), vitamin D (1000 mg daily), and vitamin E (tocopherls, 400 IU daily) were also initiated at this time to support brain, bone, and immune system development. The patient initiated formal special education instruction at 5 years of age and remains in a special education environment. Formal genetic testing was delayed as patient presentation was initially attributed to potential insult from relative prematurity, anoxia at birth, or autism spectrum disorder.

He initiated individualized neuro-optometric vision rehabilitation at age 11 years to target strabismus, binocular vision dysfunction, oculomotor dysfunction, visual–spatial dysfunction, and visual–perceptual and cognitive deficits. At baseline, his vision was not binocular, with poor spatial awareness, particularly on the z-axis in his visual space. The patient was hyper-focalized and displayed alternate fixation without simultaneous perception and could not complete basic fixation, pursuit, or saccadic tasks. He had difficulty crossing the midline, visual motor delay, and difficulty with balance. Through treatment sessions utilizing therapeutic lenses, prisms, filters, selective occlusion, eye-movement therapy, multimodal/multisensory integration, and balance therapy, he now has a functioning binocular vision system, simultaneous perception with flat fusional abilities, and intermittent stereopsis, as well as improvement in volitional and non-volitional eye movements, ability to ambulate space, hand- and eye-foot speeds, and fine motor coordination. The patient has been exposed to sports (tennis, basketball, swimming, and skiing), which have demonstrated benefit.

### 2.3. Oral Sensory Intervention

Speech and language progress, including expressive language, receptive language, and executive functioning, have been measured qualitatively. At 4 years and 10 months of age, mild oral-motor weakness persisted with praxis difficulties in alternating movement rates. Speech production was mildly dysarthric, with good ability to produce all the English phonemes except “r” and minor vowel distortions. Expressive language was marked by difficulty producing 5-step story sequences and reduced use of morphosyntactic structures with apparent agrammatism. Receptive language was marked by difficulties with following 2- to 3-step verbal commands, reduced understanding of more complex vocabulary, and generally depressed auditory processing abilities. Overall, executive functioning skills impeded language tasks, as the patient had difficulty with working memory and was highly dependent on reauditorization to execute tasks.

The patient has shown success using several therapeutic approaches to enhance education instruction provided with the common core standards. The Expanding Expression Tool (EET) has been used to improve vocabulary and metalinguistic skills. Preventing Academic Failure (PAF), a multisensory curriculum for teaching reading, spelling, and handwriting, has been useful in improving overall decoding and reading comprehension abilities. Pearson’s Cogmed Working Memory Training (CWMT) has proved beneficial in improving working memory. The patient has also improved in the domains of expressive, receptive, and executive functioning with increased opportunities for practice to enhance his understanding of core curriculum standards.

The patient is currently 15 years and 3 months of age. Oral-motor deficits are generally resolved and deemed functional. Mild praxis issues persist for volitional tasks. Speech skills are generally strong, but the patient benefits from over-articulation strategies to improve precision of vowels. Expressive language contains varied morphosyntactic structures. In conversation, he requires prompts to vary topics and can become stuck in sets. He follows directions relatively well, but visual–spatial deficits sometimes impede this ability. Reading comprehension is good when graphic organizers and note taking are used. His ability to use textual evidence to answer academic-based questions has improved significantly with faded support. Receptive language and memory are relative strengths. He continues to need assistance with organization and planning but is improving in these areas.

The patient has completed eighth grade in a special education school aligned with state core academic standards, but with an individualized education plan, individual and group speech/language and occupational therapy, and counseling. He received academic coursework scores ranging between the 84th and 94th percentiles on his most recent report card (June 2024). However, because these scores were obtained in special education instruction, they are not fully comparable to performance metrics in traditional public school. 

### 2.4. Cytogenetic Analysis

**Figure 7:**
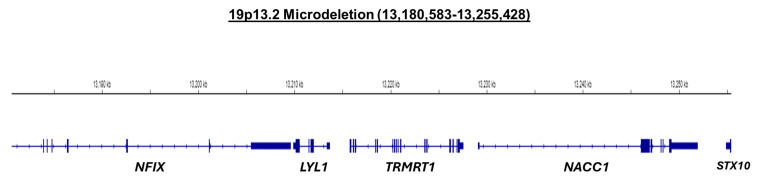
Graphical representation of the Chr 19p13.2 (13,180,583–13,255,428) deletion reported—alignment at the time of sequencing was conducted using Human (GRC37/Hg19). Image viewed from Integrative Genomics Viewer (IGV) [21].

At age 8 years, the patient underwent formal comprehensive genetic analysis. Microarray analysis, mitochondrial gene sequencing, and whole-exome sequencing revealed a normal 46, XY male karyotype with heterozygous microdeletion measuring 75 kb on the short arm of chromosome 19 at p 13.2, including exons 3 through 11 of *NFIX*. This microdeletion is likely de novo, as both parents are negative for this deletion. Whole-genome array comparative genomic hybridization (CGH) and single-nucleotide polymorphism (SNP) analysis (GeneDx, Gaithersburg, MD) revealed the following: arr [GRCh37] 19p13.2 (13,180,583–13,255,428) × 1 de novo deletion of 75 kb (Figure 7). The deleted interval contains exons 3 through 11 of *NFIX* based on transcript NM_001271043.2, a portion of *STX10*, and the entirety of *LYL1*, *TRMT1,* and *NACC1*. 

### 2.5. Neuropsychological Testing

At 19 months of age, the patient was administered the Developmental Assessment of Young Children (Table 1). The patient was diagnosed with sensory integration dysfunction, global developmental delay, and motor and oral motor apraxia. The patient also demonstrated anxiety and sensitivity to loud noises.

Additional neuropsychological testing was performed at 6, 11, and 13 years of age using the following measures: Wechsler Preschool and Primary Scale of Intelligence—Fourth Edition (preschool version for the assessment at age 6 years); Wechsler Intelligence Scale for Children—Fifth Edition; and Wechsler Individual Achievement Test—Fourth Edition for academic subtests (Table 2). The diagnostic profile across all neuropsychological evaluations remained consistent, with notable deficits in visual–spatial processing, executive functioning, and fine motor skills. Due to the nature of the patient’s cognitive challenges as well his prompt dependence, the evaluation at the age of 6 years was significantly modified to minimize interference of the test modality. These modifications were reduced in a stepwise manner for the subsequent evaluations. He was less dependent on prompts from the examiner and better able to complete tasks independently on the most recent evaluation. Thus, his performance did not accurately portray his gains or overall skill development, and gains were far more significant and clinically observable; so much so that he was able to perform several tasks that he was unable to complete in prior attempts despite modifications. Aggregate IQ was not calculated given the patient’s disparate abilities, which prevented him from completing all the subtests required to accurately calculate IQ score. Neuropsychological testing results have been provided to highlight that improvement in testing parameters continued over time.

## 3. Discussion

Overgrowth–intellectual disability syndromes represent a myriad of disorders that manifest with disorder-specific features but may be broadly associated with overgrowth, musculoskeletal abnormalities, intellectual disability, epilepsy, and mood and behavioral challenges. Genotypic and phenotypic data have been published on at least 100 patients with Malan syndrome (Table 3), the differential diagnosis for which includes Sotos, Marshall–Smith, and Weaver syndromes. These syndromes are marked by cardiac and renal anomalies, seizures, and dolichocephaly (Sotos), upper airway compromise and overall failure to thrive (Marshall–Smith) [22], and hypertelorism, dimpled chin, micrognathia, and increased incidence of malignancies, including neuroblastoma (Weaver) [23].

Phenotypically, Malan syndrome is characterized by macrocephaly, hypotonia, long and slender body habitus, long hands, and advanced bone age. Skeletal abnormalities also occur, including scoliosis, pectus carinatum/excavatum, flat occiput, long or triangular face shape, prominent forehead and frontal bossing, deeply set eyes, depressed nasal bridge, anteverted nares, down-slanted palpebral fissures, a long philtrum, thin upper vermillion in a Cupid’s bow shape, an everted lower lip, a small mouth that is often held open, and a prominent chin. Ocular abnormalities are present in 75% to 100% of patients and include strabismus, esotropia, nystagmus, poor fixation, myopia, optic nerve hypoplasia, blue sclera, and hypermetropia. Chronic diarrhea, abdominal pain, vomiting, constipation, poor feeding, malocclusion, ogival palate/overcrowded teeth, caries, oral apraxia, hypersalivation, and mitral valve regurgitation are also observed [18,20,22,24,25,26,27,28,29,30].

Brain imaging can reveal ventriculomegaly, frontal cortical atrophy, corpus callosum and vermis hypoplasia, and Chiari malformation type I [19]. Seizures, which reportedly occur in 13% to 63% of individuals with Malan syndrome, are more common in those with *NFIX* deletions compared to those with *NFIX* mutations [22,28]. Developmental delay (speech and motor apraxia) and intellectual disability are invariably present, and autistic features, anxiety, behavioral challenges, and sensitivity to noise have also been reported [20,22,24,25,26,27,28,29,30]. While there is a paucity of data on intellectual disability in patients with Malan syndrome, studies indicate that the severity can vary from mild to severe [24,25,27,28,31]. Cognitive and behavioral studies are ongoing to delineate the spectrum of intellectual disability and behavioral challenges associated with Malan syndrome.

### 3.1. Current Diagnosis, Management, and Standard of Care

Currently, there are no accepted guidelines for the diagnosis, management, or treatment of Malan syndrome. Generalized overgrowth and macrocephaly are typically the first symptoms clinically identified in patients; however, because of the significant overlap in phenotypes of overgrowth disorders, accurate diagnosis is possible only through genetic testing. Macchiaiolo and colleagues suggested a multidisciplinary approach that involves evaluation once patients are diagnosed, with appropriate follow-up performed throughout the patient’s life [18].

Consistent with other rare diseases, early identification of abnormalities and subsequent diagnosis were crucial for initiating early interventions for this patient. The intensive therapeutic support from 19 months of age has led to marked improvements in this patient’s vision, perception, fine motor skills, executive function, and language skills. However, the lack of longitudinal reports on interventions for Malan syndrome, which is largely due to the recent characterization of this syndrome, limits possible conclusions regarding the efficacy of the treatment and management protocols implemented for this patient [19]. To our knowledge, this is the first longitudinal report highlighting the clinical course, benefits of intervention, and neurocognitive development of a patient with Malan syndrome over time. Nevertheless, this report provides crucial evidence of the improvements that can be made through appropriate disease management and follow-up, the potential benefits of which should be taken into consideration for other patients. However, because these interventions treat the symptoms of the disease and not the underlying cause, there remains an ongoing need for disease-course-altering treatments that target *NFIX* directly.

### 3.2. Toward a Personalized Therapeutic Strategy

This case study presents a potential candidate for personalized therapeutic strategies such as RNA therapeutics or gene therapy. Antisense oligonucleotides (ASOs) are an attractive option in the RNA therapeutics space [32,33] and are involved in ongoing clinical trials for treatment of the neurodevelopmental disease Angelman syndrome (NCT04259281, NCT05127226), as well as the haploinsufficiency disorder Dravet syndrome (NCT04442295). In this case, the heterozygous genomic loss of *NFIX* presents as a true “haploinsufficiency model” and thus is an attractive target for the ASO-mediated upregulation of NFIX protein. However, the mechanisms by which the Angelman and Dravet syndromes ASOs operate to increase productive RNA transcripts, and consequently protein expression, are not applicable to this case due to the nature of the patient’s *NFIX* mutation. Instead, a potentially more appropriate strategy in this case is targeting ASOs to the negative regulatory elements within the 5′ untranslated region of nascent mature transcripts prior to protein translation [34,35]. By sterically blocking these regulatory elements, translation may be increased in an effort to overcome *NFIX* haploinsufficiency.

As NFIX is dose-sensitive and critically involved in the transcription of downstream genes, the extent to which is not yet known, its gross overexpression through adeno-associated virus (AAV)-mediated gene therapy could result in unwanted side effects and be deleterious. Therefore, we believe that ASOs could provide a more nuanced and regulated approach than traditional gene therapy methods [36]. It is also of note that the microdeletion observed in this case encompasses the majority of *NFIX*, as well as the entirety of *LYL1*, *TRMT1*, and *NACC1*, and a portion of *STX10*. While mutations in the non-*NFIX* genes can lead to disease, the exact role of these additional genes in the disease progression of the patient is currently unknown and warrants further molecular and preclinical testing. An example would be the ultra-rare mutation (c.892C>T) within NACC1 leading to a neurodevelopmental phenotype [37]. Preclinical studies are currently underway in reprogrammed induced pluripotent stem cells of the current case to assess the ability of ASOs to increase NFIX gene and/or protein expression.

The NFIX transcription factor plays a pivotal role in mediating stem cell proliferation, quiescence, and differentiation in multiple organ systems. NFIX loss is implicated in multiple pathogenic processes involving stem cell biology of the central and peripheral nervous systems, muscle development, hematopoiesis, and malignancy. While significant progress has been made in the last decade, work remains to elucidate the protein structure of NFIX as well as its interactions with other proteins, including members of the NFI family. Such advancements could greatly facilitate therapeutic intervention and are urgently warranted.

Since 2010, while descriptions of the distinct phenotypic features of this rare syndrome have been informative, prospective studies evaluating the cognitive, linguistic, and behavioral spectrum evinced in Malan syndrome, as well as guidance regarding therapeutic interventions to support patients, are lacking. A recent study [24] utilized various assessments (e.g., Wechsler Intelligence Scale, Leiter International Performance Scale, Griffith Mental Development Scales, among others) to assess cognitive, linguistic, and behavioral skills of 15 patients with Malan syndrome ranging from 2.7 to 25.6 years of age. Intellectual disability ranged from mild (1 of 15 patients) to severe (8 of 15 patients), and all patients had impaired linguistic skills, though language comprehension was more preserved. All patients exhibited lower than normal adaptive behavior, with the communication skills domain most affected. The case presented in this report is consistent with these assessments, but importantly highlights that a supportive therapeutic environment focused on the physical, developmental, speech, and social aspects of Malan syndrome can lead to significant improvements in function over time.

As genomic analysis techniques become more refined, we may identify a higher incidence of patients with overgrowth syndromes. Prospective analyses of therapeutic interventions in patients with overgrowth syndromes are an unmet need. Patients with Malan syndrome may benefit from robust therapeutic support, rigorous and supportive educational environments, and comprehensive preventative medical care. With the advent of personalized medicine, diseases that were previously considered untreatable, such as Duchenne muscular dystrophy and spinal muscular atrophy, now have life-altering treatment options. ASOs are continually being developed and have recently been shown to increase protein expression in animal models [34], demonstrating their promise as treatments for diseases of haploinsufficiency, such as Malan syndrome.

Given the rarity of Malan syndrome, there is limited knowledge regarding the natural history of this disorder. This case report highlights the importance of early diagnosis, early intervention, and preventative care in overgrowth syndromes, as well as the potential for therapeutic intervention in the future. It also underscores the need for improved advocacy and scientific collaboration to accelerate therapeutic discovery.

## Figures and Tables

**Table 1:** The patient’s scores on the Developmental Assessment of Young Children, administrated at 19 months of age.

Domain	Standard Score	Age-Equivalent (Months)
Cognition	72	8
Communication	66	7
Social–emotional	75	9
Physical development	58	7
Adaptive behavior	70	8

**Table 2:** The patient’s scores (by percentile) on neuropsychological testing performed at 6, 11, and 13 years of age.

	Percentile		
Skill	6 Years of Age	11 Years of Age	13 Years of Age
Verbal			
Similarities ^a^	2nd	25th	37th
Information ^b^	2nd	10th	37th
Vocabulary ^c^	CNC	CNC	37th
Visual spatial			
Block design ^d^	<1st	2nd	9th
Matrix reasoning ^e^	<1st	<1st	5th
Picture concepts ^f^	<1st	<1st	1st
Working memory			
Digits (forward) ^g^	N/A	50th	50th
Digits (backward)	N/A	CNC	5th
Digits (sequence)	N/A	5th	2nd
Picture span ^h^	N/A	5th	9th
Processing speed			
Coding ^i^	CNC	CNC	<1st
Symbol search ^j^	CNC	CNC	5th
Cancelation ^k^	CNC	CNC	<1st
Reading			
Word identification	N/A	50th	50th
Word comprehension	N/A	25th	25th
Pseudoword reading	N/A	10th	30th
Orthographic fluency	N/A	CNC	27th
Comprehension	N/A	37th	25th
Math			
Numerical operations	CNC	9th	25th
Problem solving	CNC	1st	16th
Math fluency	N/A	7th	12th
Writing			
Spelling	CNC	20th	30th
Writing fluency	N/A	6th	58th
Sentence combining	N/A	CNC	3rd
Sentence building	N/A	CNC	25th
Essay composition	N/A	CNC	4th

Tests administered include the Wechsler Preschool and Primary Scale of Intelligence—Fourth Edition (preschool version used at age 6 years); Wechsler Intelligence Scale for Children—Fifth Edition; and Wechsler Individual Achievement Test—Fourth Edition. ^a^ Similarities: compare how 2 things are categorically related. ^b^ Information: answer general knowledge questions. ^c^ Vocabulary: give a definition to a word. ^d^ Block design (timed): assemble blocks to match a target design. ^e^ Matrix reasonings: solve for a missing visual pattern. ^f^ Picture concepts: pick a picture from each row that shares a common characteristic. ^g^ Digit span: repeat a set of numbers that have been read aloud forwards, backwards, and in ascending order. ^h^ Picture span: recall a set of pictures after a 5 s exposure. ^i^ Coding (timed): use an answer key to copy symbols that match a number code. ^j^ Symbol search (timed): scan a line of symbols and identify duplicates in each row. ^k^ cancelation (timed): cross out a set of specific pictures that appear on a page of many pictures. Abbreviations: CNC, could not complete; N/A, not applicable.

**Table 3:** Phenotypic characteristics observed among individuals with Malan syndrome *.

Characteristic	Percentage of Individuals
Macrocephaly	>75%
Hypotonia	50–76%
Long and slender body habitus	59–100%
Long hands	63%
Advanced bone age	80%
Scoliosis	32–75%
Pectus carinatum/excavatum	40–56%
Flat occiput, long or triangular face shape	81%
Prominent forehead and frontal bossing	100%
Deeply set eyes	50%
Depressed nasal bridge	50%
Anteverted nares	69%
Down-slanted palpebral fissures	63%
Long philtrum	56%
Thin upper vermillion in a Cupid’s bow shape	75%
Everted lower lip	81%
Small mouth that is often held open	69%
Prominent chin	88%
Ocular abnormalities	75–100%
Strabismus	32–63%
Esotropia	≤56%
Nystagmus	15–31%
Optic nerve hypoplasia	21–25%
Blue sclera	25–69%
Constipation	50%
Malocclusion	44%
Ogival palate/overcrowded teeth	2–56%
Caries	38%
Hypersalivation	31%
Mitral valve regurgitation	1–31%
Ventriculomegaly	27–50%
Corpus callosum and vermis hypoplasia	22–50%
Chiari malformation type I	9.5–38%
Seizures	13–63%
Intellectual disability	100%
Autistic features	31%
Sensitivity to noise	81%

* Data reported in this table have been collated from the following sources: Alfieri et al. 2022 [24]; Bellucco et al. 2019 [25]; Dolan et al. 2010 [20]; Gurrieri et al. 2015 [26]; Klaassens et al. 2015 [22]; Kuroda et al. 2017 [27]; Macchiaiolo et al. 2022 [18]; Malan et al. 2010 [19]; Mulder et al. 2020 [28]; Priolo et al. 2018 [29]; Yoneda et al. 2012 [30].

## Data Availability

The data that support the findings of this study are available on request from the corresponding author. The data are not publicly available due to their containing information that could compromise the privacy of the patient.
